# Size and Illumination Matters: Local Magnetic Actuation and Fluorescence Imaging for Microrobotics

**DOI:** 10.1007/s41745-024-00453-5

**Published:** 2025-02-10

**Authors:** Juan J. Huaroto, Sarthak Misra

**Affiliations:** 1 Surgical Robotics Laboratory, Department of Biomechanical Engineering, University of Twente, 7522 NB, Enschede, The Netherlands; 2 Surgical Robotics Laboratory, Department of Biomaterials and Biomedical Technology, University Medical Centre Groningen and University of Groningen, 9713 GZ, Groningen, The Netherlands

**Keywords:** Microrobotics, Surgical instruments, Miniaturized electromagnets, Fluorescence imaging, Two-photon microscopy.

## Abstract

Combining local magnetic actuation with fluorescence imaging modalities promises to introduce significant advances in microrobotic-guided procedures. This review presents the advantages and challenges of this approach, emphasizing the need for careful design considerations to optimize performance and compatibility. Traditional microrobotic actuation systems rely on bulky electromagnets, which are unsuitable for clinical use due to high power requirements and limited operational workspace. In contrast, miniaturized electromagnets can be integrated into surgical instruments, offering low power consumption and high actuation forces at the target site. Fluorescence imaging modalities have been explored in microrobotics, showcasing spatiotemporal resolution and the capability to provide information from biological entities. However, limitations, such as shallow penetration depth and out-of-focus fluorescence, have motivated the development of advanced techniques such as two-photon microscopy. The potential of two-photon microscopy to overcome these limitations is highlighted, with supporting evidence from previous studies on rat tissue samples. Current challenges in optical penetration depth, temporal resolution, and field of view are also addressed in this review. While integrating miniaturized electromagnets with fluorescence imaging modalities holds the potential for microrobotic-guided procedures, ongoing research and technological advancements are essential to translating this approach into clinical practice.

## Introduction

In recent years, the field of microrobotics has witnessed significant advancements driven by the demand for precise manipulation and imaging at the microscale^1–5^. Microrobots or micro-agents serve as essential tools for navigating through intricate media, facilitating applications across various domains, including targeted drug delivery^6^, cell manipulation^7^, and minimally invasive surgery^8^. With the ongoing progress in microrobotic fabrication and functionalization, efficient actuation methods and imaging modalities are crucial to unlocking novel applications and expanding the application domain toward clinically relevant scenarios.

Micro-agents can be engineered to respond to external stimuli such as magnetic fields^9–11^, acoustic waves^12–14^, and light^15–17^. In particular, magnetic actuation has emerged as a promising approach for propelling and maneuvering micro-agents in a contactless fashion^18^. Due to their transparency and biocompatibility, magnetic actuation has been utilized for micro-agent navigation in complex environments, achieving groundbreaking applications under *ex vivo* and *in vivo* conditions^19^. By leveraging magnetic fields and gradients, magnetic actuation systems allow precise control without needing onboard power sources or complex mechanical components. In the present-day context, several magnetic actuation platforms have emerged following the use of bulky structures assembled to permanent magnets, electromagnets, and robotic platforms^20–22^. Motivated by the need to reduce the size and power consumption of magnetic systems, prior research has introduced the concept of local actuation via miniaturized magnets^23–29^. Miniaturized magnets provide direct access to target regions due to their size and potential integration into surgical instruments for in situ actuation of micro-agents.

In parallel with advancements in magnetic actuation, visualizing and tracking micro-agents in real-time is essential for understanding their dynamics and validating their interaction with biological entities^30–33^. Various imaging modalities have been proposed and adapted to visualize micro-agents to optimize spatiotemporal resolution, penetration depth, and the capability to obtain molecular information from living cells^34–36^. Among non-ionizing standard modalities such as magnetic resonance, photoacoustics, and ultrasound, fluorescence-based imaging techniques provide a powerful means of monitoring micro-agents with a relatively high spatiotemporal resolution but limited penetration depth^37,38^. Fluorescence imaging is an appealing modality for visualizing micro-agents alongside biological entities, permitting the study of the response to external stimuli with remarkable sensitivity^7,39,40^. Advanced fluorescence modalities such as two-photon microscopy are available in the literature to overcome the limited penetration depth of traditional fluorescence imaging^41^. However, additional efforts are required to bridge the gap between two-photon microscopy and microrobotics^42,43^. Other techniques, using higher-order photon absorption (e.g., three-photon microscopy)^44^ and actively adjusting phase and amplitude of light wavefront (i.e., wavefront shaping)^45^, are promising for breaking the scattering limits of light in biological media. Nevertheless, the literature has not explored their integration into the microrobotics field.

The convergence of local magnetic actuation and fluorescence imaging in microrobotics presents an opportunity to unlock clinical advancements. The unique capabilities of fluorescence imaging can be used for precise micro-agent tracking while offering insights into biological environments. Moreover, miniaturized magnets facilitate their integration into minimally invasive surgical instruments, enabling in situ micro-agent control. Merging both technologies represents an advancement in microrobotics, accelerating progress in research and clinical applications.

### Scope and Outline

This review presents the current progress on magnetic actuation and fluorescence-based imaging to control and monitor micro-agents. Throughout the review, we present the types of magnetic actuation systems, emphasizing miniaturized electromagnets for local actuation of micro-agents. We discuss the need for advanced imaging modalities to achieve micro-agent in-tissue control. We highlight the principles of fluorescence imaging, its application to the microrobotics field, and current challenges in minimally invasive surgery. By combining local magnetic actuation and fluorescence imaging, we seek to address fundamental challenges in microrobotics, including precise manipulation in confined spaces and tracking within biological environments.

The remainder part of the review is divided into four sections. First, we summarize the current magnetic actuation systems, introduce the concept of local actuation, and present the equations governing microrobotic actuation using miniaturized electromagnets integrated into two types of minimally invasive surgical instruments. Second, we introduce fluorescence imaging for microrobotics, discuss the limitations of traditional modalities, and propose advanced techniques, such as two-photon microscopy, to address these shortcomings. Third, we explore the integration of miniaturized electromagnets and two-photon microscopy to enable fluorescence-guided micro-agent manipulation. Finally, we summarize and discuss the concepts presented throughout the review.

## Electromagnetic Actuation in Microrobotics

### Background

**Figure 1:**
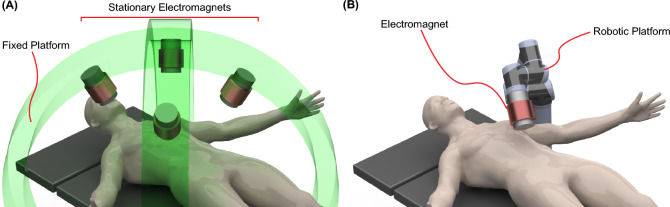
Electromagnetic systems located outside the human body: Traditional arrangements. **A** Electromagnets assembled in a fixed platform. **B** Electromagnets assembled at the end effector of a robotic platform.

Magnetic actuation has been studied to enable contactless manipulation of micro/milli-robotic structures and advanced catheters to access hard-to-reach regions of the human body^9,21^. In particular, the apparatus used in microrobotics has constantly evolved to address application-oriented requirements^20,22,46,47^. Permanent magnets and electromagnets are standard devices used in magnetic actuation systems to generate magnetic fields and gradients. Permanent magnets allow for the precise generation of magnetic fields at a defined distance from the magnet. However, the generation of time-varying magnetic fields is limited, necessitating robotic platforms to position or spin the permanent magnet with respect to a target region or workspace^48–53^. Unlike permanent magnets, electromagnets or electromagnetic coils can generate magnetic fields and gradients by powering them with electrical currents. In electromagnetic actuation systems, electromagnets can be arranged in stationary mechanical structures^54–59^ (Fig. 1A) and robotic structures^59–62^ to span a desired workspace (Fig. 1B).

In clinically relevant scenarios, electromagnetic systems are typically designed to be positioned externally, enabling non-invasive actuation of micro-agents inside the body. Traditional electromagnetic systems have successfully demonstrated medical applications using *in vivo* animal samples or human cadavers^63–66^. However, these systems are accompanied by various challenges that must be addressed to advance their translation to clinical settings. The magnetic field required to propel micro-agents within vessels must overcome physiological fluids to reach a desired region^67^. In practice, addressing the generation of relatively high magnetic fields and gradients often involves increasing the electromagnet size, adjusting electrical currents, or adding additional electromagnets to the actuation system^68^. However, the power consumption and space limitations of surgical rooms slow down the standardization of electromagnetic systems for human trials.

### Miniaturized Systems

**Figure 2:**
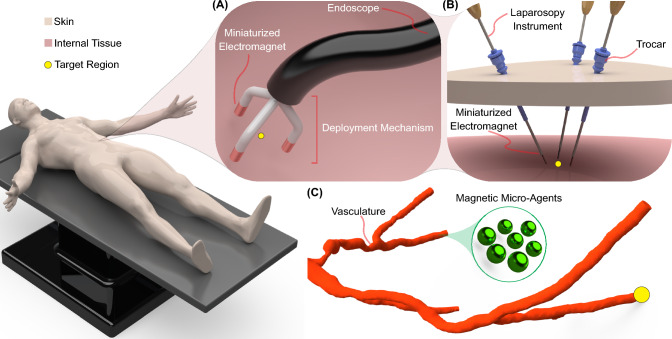
Miniaturized electromagnets integrated into surgical instruments to perform local actuation of micro-agent. **A** Endoscope with miniaturized electromagnets deployed around a target region. **B** Laparoscopic instruments with integrated needle-shaped electromagnets. The trocars permit the manipulation of the instruments to span the surroundings of a target region. **(C)** Zoom in on the workspace showing magnetic micro-agents being pulled within a vasculature toward a target region.

A different approach to addressing the challenges of traditional electromagnetic systems for microrobotics involves the integration of miniaturized electromagnets into surgical instruments such as endoscopes^25^ and laparoscopy probes^26^ (Fig. 2). This integration offers the advantage of minimal invasiveness, making it a promising alternative in microsurgery where precise manipulation is required^6^. Compared with traditional electromagnetic systems, miniaturized electromagnets can significantly reduce power consumption (by three orders of magnitude) and provide direct access to biological workspaces while maintaining biologically compatible temperatures. Moreover, the magnetic field gradients near miniaturized electromagnets are comparable to the values registered at the workspace center of electromagnetic systems (Table 1).

**Table 1.** Magnetic field $\left( ||\textbf{B}||\right)$ and gradient-to-field ratio $\left( \frac{||\nabla \textbf{B}||}{||\textbf{B}||}\right)$ of external and local electromagnetic actuation systems. Table adapted from^28^

Configuration	Actuation system	Moving electromagnets	$||\textbf{B}||$ $\text {[mT]}$	$||\mathbf {\nabla B}||/||\textbf{B}||$ $\text {[m}^{-1}\text {]}$
External	Open Configuration ^57^	No	15	35
	Antiprism ^55^	No	30	40
	OctoMag ^54^	No	30	25
	Toroidal ^69^	No	50	5
	Maxwell ^70^	No	100	15
	BatMag ^58^	No	100	20
	CGCI ^68^	No	100	8
	Orthogonally Aligned ^56^	No	40	6
	DeltaMag ^62^	Yes	23	–
	BigMag ^60^	Yes	40	25
	ARMM ^61^	Yes	80	7
Local	MiniMag ^71^	No	20	100
	MILiMAC ^25^	No	8	370
	MagNeed ^26^	No	9	380
	µMAZE ^24^	No	0.15	8000

Integrating miniaturized electromagnets into surgical instruments can revolutionize surgical procedures and introduce new treatments using magnetic micro-agents^25,26^. Previous studies have proposed such integration to provide basic functionalities, such as gripping small objects^27,29^. However, local actuation of micro-agents using miniaturized electromagnets presents challenges, requiring careful consideration of electric and thermal insulation. Additionally, unique strategies for the deployment and retrieval of miniaturized electromagnets need to be developed and tailored to surgical instruments.

For endoscopes, miniaturized electromagnets can be delivered through an internal channel and positioned around a target region using a deployment mechanism (Fig. 2A). This mechanism is deactivated when micro-agents reach a target region, permitting retrieval through the endoscope channel^25^. Typically, the deployment mechanism must be a small apparatus activated/deactivated using external stimuli such as mechanical force transmitted through cables^72^, magnetic fields^73^, or temperature gradients to trigger the shape memory effect of advanced materials^74,75^.

For laparoscopy probes, miniaturized electromagnets can be designed in the shape of needles to facilitate their assembly^26^. Unlike endoscopes, electromagnet deployment and retrieval are achieved using standard pivot points, such as trocars^76^. The advantage of using these pivots is that the spatial configuration of the probes can be actively modified to change the workspace configuration (Fig. 2B). Furthermore, laparoscopy probes with assembled electromagnets can be attached to robotic platforms to enable object avoidance and image-guided procedures^77,78^.

### Fundamentals

In order to understand the local actuation of micro-agents using miniaturized electromagnets, we present the fundamentals of actuation through mathematical formulation. We utilize three miniaturized electromagnets throughout the analysis to achieve 3D manipulation^79^. However, the formulation can be extended to include more electromagnets, creating an overactuated system.

In general, the magnetic field generated by an electromagnet $\left( \textbf{B}(\textbf{p},I)\right)$ powered with an electrical current ($I \in \mathbb {R}$) exerts a wrench ($\textbf{W} \in \mathbb {R}^6$) over a micro-agent with magnetic moment ($\varvec{\mu } \in \mathbb {R}^3$) located at a point ($\textbf{p} \in \mathbb {R}^3$). The magnetic wrench includes the magnetic force ($\textbf{F} \in \mathbb {R}^3$) and magnetic torque ($\textbf{T} \in \mathbb {R}^3$) and is defined as

1 $$\begin{aligned} \textbf{W}(\textbf{p}) = \begin{bmatrix}\textbf{F}(\textbf{p}) \\ \textbf{T}(\textbf{p})\end{bmatrix} = \begin{bmatrix}\nabla (\varvec{\mu }\cdot \textbf{B}(\textbf{p},I)) \\ \varvec{\mu }\times \textbf{B}(\textbf{p},I)\end{bmatrix}. \end{aligned}$$

**Figure 3:**
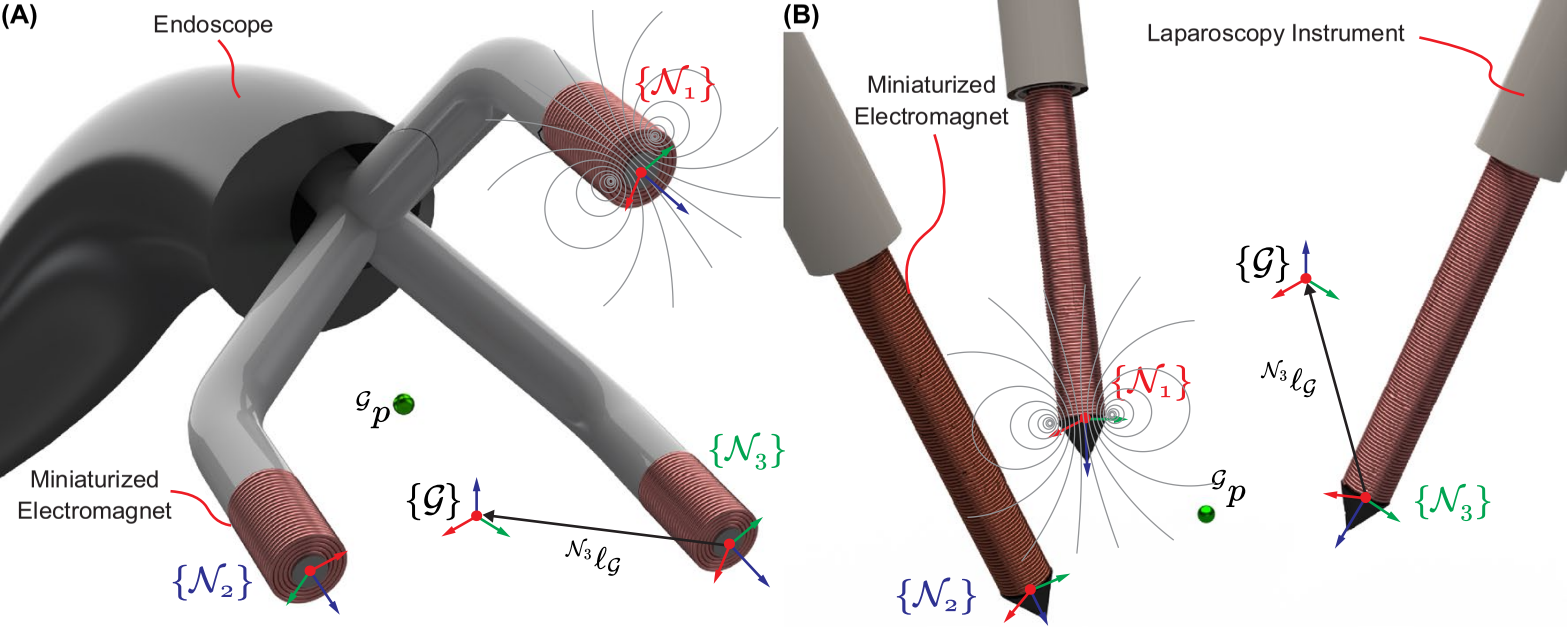
Reference frames used to mathematically describe the actuation of a magnetic micro-agent located at point $\left( ^{\mathcal {G}}\textbf{p}\right)$. **A** Endoscope with integrated miniaturized electromagnets. **B** Laparoscopy instruments with integrated needle-shaped electromagnets. The local frames attached to each electromagnet $\left( \{\mathcal {N}_k\}\text {, for }k\,\text {= 1, 2, 3}\right)$ are constructed with respect to the global frame $\left( \{\mathcal {G}\}\right)$. The distances between local and global frames are $^{\mathcal {N}_k}\varvec{\ell }_{\mathcal {G}}$.

Local frames $\left( \{\mathcal {N}_k\}\text {, for }k\,\text {= 1, 2, 3}\right)$ are constructed for three miniaturized electromagnets with respect to a global reference frame $\left( \{\mathcal {G}\}\right)$ (Fig. 3). At a point, $\left( ^{\mathcal {G}}\textbf{p} \in \mathbb {R}^3\right)$ in the global reference frame, the magnetic field $\left( ^{\mathcal {G}}\textbf{B}(^{\mathcal {G}}\textbf{p})\right)$ is the sum of the magnetic field generated by each electromagnet $\left( ^{\mathcal {G}}\textbf{B}_k(^{\mathcal {G}}\textbf{p})\right)$. The unitary magnetic field $\left( ^{\mathcal {N}_k}\varvec{\beta }_k(^{\mathcal {N}_k}\textbf{p})\right)$ is generated in the local frame of the corresponding electromagnet. The miniaturized electromagnets operate in their linear regions (i.e., the magnitude and components of the magnetic field vary linearly with the current)^80^. Hence, the magnetic field in local frames is defined as

2 $$\begin{aligned} ^{\mathcal {N}_k}\textbf{B}_k(^{\mathcal {N}_k}\textbf{p})={^{\mathcal {N}_k}\varvec{\beta }_k(^{\mathcal {N}_k}\textbf{p})}I_k, \end{aligned}$$

where $I_k \in \mathbb {R}$, is the current through the *k*th electromagnet. In order to compute the vectors $^{\mathcal {G}}\textbf{B}_k(^{\mathcal {G}}\textbf{p})$, we define the rotation matrices $\left( ^{\mathcal {G}}\varvec{R}_{\mathcal {N}_k} \in SO(3)\right)$. This way, $^{\mathcal {N}_k}\textbf{p} = {^{\mathcal {N}_k}\varvec{R}_{\mathcal {G}}}\,^{\mathcal {G}}\textbf{p} + {^{\mathcal {N}_k}\varvec{\ell }_{\mathcal {G}}}$, where $^{\mathcal {N}_k}\varvec{\ell }_{\mathcal {G}} \in \mathbb {R}^3$ are the distance vectors between local and global frames (Fig. 3). Hence, $^{\mathcal {G}}\textbf{B}_k(^{\mathcal {G}}\textbf{p})$ is computed as follows:

3 $$\begin{aligned} ^{\mathcal {G}}\textbf{B}_k(^{\mathcal {G}}\textbf{p})={^{\mathcal {G}}\varvec{R}_{\mathcal {N}_k}}{^{\mathcal {N}_k}\varvec{\beta }_k(^{\mathcal {N}_k}\textbf{p})}I_k. \end{aligned}$$

Substituting (3) into (1), we obtain the magnetic wrench $\left( ^{\mathcal {G}}\textbf{W}(^{\mathcal {G}}\textbf{p})\right)$ composed of force $\left( ^{\mathcal {G}}\textbf{F}(^{\mathcal {G}}\textbf{p})\right)$ and torque $\left( ^{\mathcal {G}}\textbf{T}(^{\mathcal {G}}\textbf{p})\right)$ exerted on a micro-agent located at $^{\mathcal {G}}\textbf{p}$:

4 $$\begin{aligned} {\left\{ \begin{array}{ll} ^{\mathcal {G}}\textbf{F}(^{\mathcal {G}}\textbf{p}) = \varvec{\mu }^T \begin{bmatrix}\frac{\partial \left[ {^{\mathcal {G}}\varvec{\beta }_1(^{\mathcal {G}}\textbf{p})}\right] }{\partial x}\,\dots \,\frac{\partial \left[ {^{\mathcal {G}}\varvec{\beta }_3(^{\mathcal {G}}\textbf{p})}\right] }{\partial x}\\ \frac{\partial \left[ {^{\mathcal {G}}\varvec{\beta }_1(^{\mathcal {G}}\textbf{p})}\right] }{\partial y}\,\dots \,\frac{\partial \left[ {^{\mathcal {G}}\varvec{\beta }_3(^{\mathcal {G}}\textbf{p})}\right] }{\partial y}\\ \frac{\partial \left[ {^{\mathcal {G}}\varvec{\beta }_1(^{\mathcal {G}}\textbf{p})}\right] }{\partial z}\,\dots \,\frac{\partial \left[ {^{\mathcal {G}}\varvec{\beta }_3(^{\mathcal {G}}\textbf{p})}\right] }{\partial z}\end{bmatrix}\begin{bmatrix}I_1\\ I_2\\ I_3\end{bmatrix}.\\ ^{\mathcal {G}}\textbf{T}(^{\mathcal {G}}\textbf{p}) = \varvec{\mu } \times \sum \limits _{k = 1}^{3}\left( ^{\mathcal {G}}\textbf{B}_k(^{\mathcal {G}}\textbf{p})\right) \end{array}\right. } \end{aligned}$$

The presented formulation utilizes the spatial configuration of local frames $\left( \{\mathcal {N}_k\}\right)$ and the set of currents ($I_k$) as inputs to compute equations (2)–(4) in every actuation period. Considering the characteristics of instruments integrating miniaturized electromagnets, constraints can be applied to local frames to reduce computational complexity. In the case of an endoscope integrating such electromagnets, it is reasonable to assume that the deployment mechanism will fix the electromagnets around a target region. Consequently, local frames remain approximately stationary, and the magnetic field and gradients at point $\left( ^{\mathcal {G}}\textbf{p}\right)$ are solely determined by the currents (Fig. 3A). For laparoscopy instruments integrating miniaturized electromagnets, the local frames can change their pose according to the instrument’s motion (Fig. 3B). Hence, rotation matrices $\left( ^{\mathcal {G}}\varvec{R}_{\mathcal {N}_k}\right)$ and vector distances $\left( ^{\mathcal {N}_k}\varvec{\ell }_{\mathcal {G}}\right)$ are updated in every actuation period. In order to ensure accuracy and robustness and account for external disturbances, various algorithm schemes for micro-agent control can be implemented for miniaturized electromagnets^81^. Hence, the development of clinical imaging modalities is necessary to ensure the required feedback for precise manipulation of micro-agents.

## Fluorescence Imaging for Microrobotics

### Background

Visualization or imaging is essential in microrobotics because current sensor technology cannot be incorporated into micro-agents due to size limitations. Imaging modalities serve as external sensors to validate micro-agent functionalities and provide feedback during procedures. Recent research in the field of microrobotics underscores the significance of advanced methods in broadening the scope of microrobotics into clinically relevant scenarios^82–85^. Microrobotics imaging requires three main components to detect and track micro-agents under *in vivo* conditions.

- **Spatiotemporal resolution and penetration depth:** Imaging modalities in microrobotics must enable micrometer resolution, video-rate tracking, and sufficient penetration depths to achieve in-tissue visualization^35^. Generally, a trade-off exists between spatial resolution and penetration depth due to tissue scattering. Therefore, an optimal balance between spatiotemporal resolution and penetration depth is essential for in-tissue microrobotic imaging.
- **Molecular imaging:** Visualization modalities must ensure the detection of micro-agents and the surrounding physical environment, including tissue and biological samples^38^. Studying living cells, biological processes, and tissue interacting with functionalized micro-agents requires addressing molecular imaging techniques^36^.
- **Non-ionizing modalities:** Prolonged exposition to ionizing ration risks side effects on patients and clinicians^86^. Non-ionizing modalities can enable safe procedures using micro-agents.

Current imaging modalities for microrobotics partially address all three components. Various ionizing techniques such as X-ray computerized tomography^65^, positron emission tomography^87^, fluoroscopy^88^, and single-photon emission computed tomography^89^ have been proven under *ex vivo* and *in vivo* conditions. Yet, the risk of side effects from long-term exposure motivates the use of non-ionizing techniques. Among non-ionizing modalities, fluorescence imaging^38^, ultrasound^31^, magnetic resonance imaging^90^, photoacoustic imaging^84,91^, and magnetic particle imaging^92^ provide molecular information, revealing details about the interaction between micro-agents, biological entities, and living tissue. This review focuses on fluorescence imaging modalities and their application in microrobotics. Compared to other non-ionizing imaging techniques, the relatively high spatiotemporal resolution of fluorescence imaging makes it attractive for studying biological processes in clinical settings using microrobots^35^. However, a major limitation of fluorescence imaging is the restricted optical penetration depth caused by tissue scattering. Advanced fluorescence imaging modalities using high order absorption such as two- and three-photon microscopy^93^ and tuning the phase and amplitude of the light wavefront (i.e., wavefront shaping)^94^ represent state-of-the-art techniques for overcoming the scattering limits of tissue. Nonetheless, further efforts are required to integrate these techniques into the field of microrobotics.

### Fluorescence

Fluorescence is a phenomenon in which a substance (fluorescent dye or fluorophore) absorbs electromagnetic radiation at one wavelength and then emits light at a longer wavelength^95^. This shift between wavelengths is called Stokes shift and represents the difference in wavelength between the maximum excitation ($\lambda _{\text {ex}}$) and maximum emission ($\lambda _{\text {em}}$) of a fluorophore:

5 $$\begin{aligned} \text {Stokes Shift} = \lambda _{\text {ex}} - \lambda _{\text {em}}. \end{aligned}$$

In fluorescence, the emission occurs promptly after excitation, often within nanoseconds to microseconds^96^. This property distinguishes fluorescence from other types of photoluminescence, such as phosphorescence, where the emission persists for a longer duration after excitation. Spectrum analysis using spectrofluorometers provides intensity-wavelength plots to study the excitation and emission light characteristics of fluorophores^97^. The fluorescence quantum yield ($\Phi$) measures the efficiency of a fluorophore to emit fluorescence upon excitation:

6 $$\begin{aligned} \Phi = \frac{\text {Number of photons emitted}}{\text {Number of photons absorbed}}. \end{aligned}$$

The value of $\Phi$ depends on the fluorophore and solvents utilized^98^. Besides, the intensity of emitted fluorescence light is proportional to the number of fluorophores in the sample and quantum yield.

**Figure 4:**
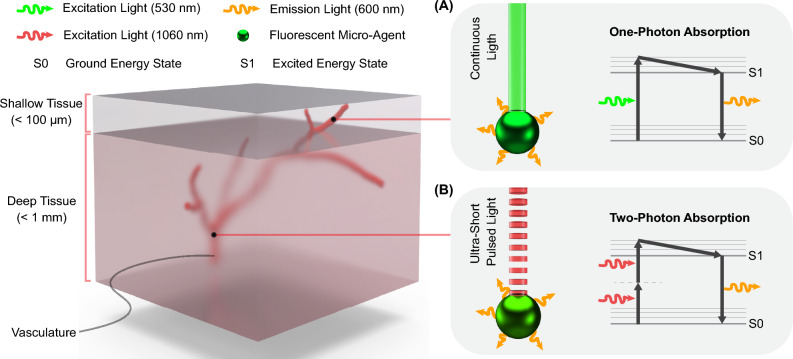
Fluorescence imaging for shallow and deep tissue. **A** Conventional fluorescence imaging utilizes the principle of one-photon excitation (excitation light at 530 nm wavelength) to generate emission light at 600 nm wavelength. One-photon excitation can be used for shallow tissue as it is prone to background noise and scattering. **B** Two-photon microscopy is an advanced fluorescence imaging modality that uses the principle of two-photon excitation. As two photons are absorbed simultaneously, the excitation light is a femtosecond-pulsed laser with 1060 nm wavelength. By using near-infrared light, the light can penetrate tissue samples deeper. Besides, the two-photon excitation only occurs at the focal spot, eliminating the background noise.

Fluorescence is widely utilized in various fields, including biology, chemistry, and surgery, for diagnostics and microscopy applications^99,100^. Due to their specificity, sensitivity, and versatility, fluorophores are commonly employed to label biological samples, track molecular processes, and functionalize micro-agents^40^. In microscopy, the excitation light is focused onto the sample, and the emitted light is collected through lenses and a series of dichroic mirrors, which redirect the light for image formation through detectors (e.g., complementary metal-oxide semiconductor cameras and photomultipliers)^93,101^. Figure 4A depicts a fluorescent micro-agent emitting fluorescence with a wavelength of 600 nm under excitation by a continuous light source of 530 nm. The energy diagram, including ground and excited energy states, represents a fluorescence event in which a single photon at 530 nm is absorbed to generate one photon of emitted light at 600 nm. This energy representation corresponds to one-photon absorption and is the functioning principle of many fluorescence microscopes used in biology. Fluorescence imaging modalities are the gold standard for color-coded imaging using different fluorophores to label biological samples and micro-agents^7,38^. However, the limited optical penetration depth, scattering, and background noise reduce the capability of using conventional fluorescence microscopes to visualize micro-agent in-tissue.

### Two-Photon Microscopy

An advanced fluorescence modality to overcome the limited penetration depth of fluorescence imaging techniques is two-photon microscopy^41,102^. Unlike conventional fluorescence microscopy, two-photon microscopy utilizes the simultaneous absorption of two lower-energy photons to excite fluorophores within a specimen (Fig. 4B). In two-photon microscopy, an ultrashort-pulsed laser emits near-infrared (NIR) light photons, typically within 700–1100 nanometers of wavelength. This longer wavelength light can penetrate deeper into biological tissues with minimal scattering and absorption, allowing imaging of thicker samples or even intact living organisms^103^.

When two photons of NIR light coincide in space and time, their combined energy excites a fluorophore to its higher energy state^104^. This excitation occurs only at the focal point of the microscope, where the intensity of the laser light is highest, resulting in precise spatial localization of fluorescence emission^105,106^. Because fluorescence is generated only at the focal point, out-of-focus fluorescence (i.e., background noise) is minimized, improving image contrast and resolution, especially in thick samples. Two-photon microscopy offers three essential advantages over traditional fluorescence microscopy techniques, including:

- **Deep tissue imaging:** The longer wavelength of NIR light allows deeper penetration into biological specimens, enabling imaging of structures located several hundred micrometers below the tissue surface.
- **Reduced photodamage:** Because fluorescence excitation occurs only at the focal point, photodamage to the specimen outside the focal plane is minimized, making two-photon microscopy suitable for imaging living tissues over extended periods without significant damage.
- **Increased image contrast and resolution:** By minimizing out-of-focus fluorescence, two-photon microscopy provides improved image contrast and resolution, particularly in thick or densely labeled samples.
- **Fluorophore-free imaging:** The ultrashort-pulsed laser used in two-photon microscopy can trigger autofluorescence and second-harmonic generation in biological tissue samples^107^. This way, fluorophore-free imaging is enabled, eliminating photobleaching effects associated with standard fluorophores^108^.

Two-photon microscopy is widely used in neuroscience, cell biology, and other fields where high-resolution imaging of living tissues or organisms is essential^109,110^. In addition, various endomicroscopy probes have been developed to diagnose and study cellular processes in freely behaving animals^111^. In microrobotics, the principle of two-photon absorption has mainly been used to fabricate sub-micrometer resolution structures such as lenses^112^, sensors^113^, micro-agents^114^, and metamaterials^115^. Two-photon microscopy recently garners attention using benchtop microscopes to visualize micro-agents through *ex vivo* and *in vivo* rat tissue^42,43^.

### Open Challenges

Although fluorescence imaging can provide information from biological samples and tissue, the optical penetration depth remains a challenge due to tissue scattering. Two-photon microscopy has demonstrated clinical applications for diagnosis and revolutionized our understanding of biological processes by enabling the visualization of cellular dynamics and interactions with unprecedented detail^93,116^. However, the optical penetration depth cannot exceed hundreds of micrometers. Using three-photon absorption has opened up new avenues for imaging tissue at penetration depths on the order of 1 mm in brain tissue^117,118^. Increasing the order of photon absorption enables greater penetration depth due to the longer wavelength used to trigger fluorescence. However, additional challenges must be addressed, such as the limited field of view, temporal resolution, and chromatic dispersion^111^.

On the other hand, wavefront shaping offers an innovative approach to overcoming the scattering limits of tissue. This technique modifies the phase and amplitude of light, enabling deep focusing through scattering media up to tens of centimeters^94,119–121^. The convergence of high-order photon absorption and wavefront shaping can alleviate the limited penetration depth of fluorescence microscopy. However, fluorescence collection at relatively high penetration depths is an open challenge due to the absorption probability of emitted photons in scattering media. A comprehensive study of the algorithms to reconstruct images from fluorescence-emitted light in deep tissue is required^45,122^.

## Toward Fluorescence-Guided Manipulation

**Figure 5:**
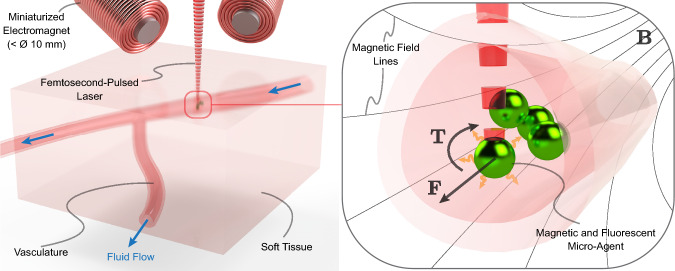
Illustration of fluorescence-guided manipulation of functionalized micro-agents. The magnetic micro-agents are within a vasculature transporting fluid flow. Miniaturized electromagnets are used to actuate micro-agents, while two-photon microscopy is used to obtain feedback. The excitation light (i.e., femtosecond-pulsed laser) penetrates tissue and vasculature to trigger emission light from fluorescent micro-agents. The magnetic field ($\textbf{B}$) generated by the electromagnets exerts a force ($\textbf{F}$) and torque ($\textbf{T}$) over the micro-agent.

**Figure 6:**
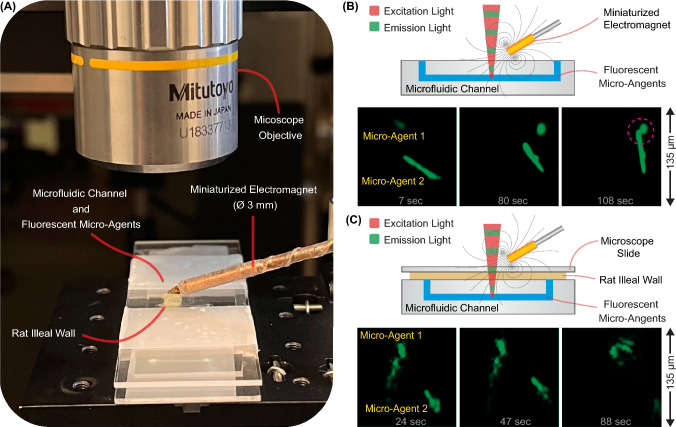
Fluorescence-guided manipulation of micro-agents (magnetic electrospun fibers). **A** Experimental validation platform for two-photon image acquisition of micro-agents through rat tissue. The micro-agents are attached due to the electrostatic interaction. **B** Visualization of micro-agents within a microfluidic channel. The pink dashed circle indicates attachment due to electrostatic interaction. **C** Visualization of micro-agents through rat tissue. Figure adapted from^42^.

The convergence of surgical instruments integrating miniaturized electromagnets and fluorescence imaging modalities can improve the precision and efficiency of targeted drug delivery procedures via functionalized micro-agents. By overcoming physiological fluid flows and scattering, fluorescence-guided manipulation of magnetic micro-agents represents a groundbreaking approach to advancing the application of microrobotics in clinically relevant scenarios. Figure 5 depicts a tissue segment containing vasculature. The micro-agents previously perfused within the vasculature are actuated and visualized through tissue using miniature electromagnets and two-photon microscopy, respectively. Our previous study demonstrates this approach using formalin-fixed rat intestinal tissue (ileal wall)^42^. Figure 6A shows a close-up view of the experimental setup, including a miniaturized electromagnet^26^. Magnetic electrospun fibers stained with a fluorophore (Coumarin 6) are fabricated as the micro-agents used in the experiments^38^. Micro-agents are perfused within a microfluidic channel to visualize their interactions through two-photon microscopy. For comparison, we used two types of samples: a plain microfluidic channel (Fig. 6B) and a microfluidic channel containing a slice of rat tissue (thickness $\approx$ 600 µm) (Fig. 6C).

Using the first sample enables clear visualization of the orientation and motion of micro-agents in response to the magnetic field and gradients produced by the miniaturized electromagnet (Fig. 6B). The experiment utilizing formalin-fixed rat tissue intensifies scattering, augmenting the complexity of visualizing micro-agents. Despite the scattering challenges, our results show that two-photon microscopy facilitates the identification of morphology and tracking of micro-agents’ motion while enabling continuous image acquisition.

It is worth noting that the experiments were achieved using a relatively low frame acquisition rate (1 fps), which can limit the analysis of micro-agents moving at high dynamics. Previous *in vivo* studies demonstrate that micro-agents can move at relatively low velocities (< 10 µm/s) under magnetic guidance^83,123^. Furthermore, incorporating optical and optomechanical technologies, such as polygonal mirrors and resonant scanners coupled with optical fibers, can drastically improve the scan rate by compromising the spatial resolution^116,124^. On the other hand, the field of view used for image acquisition is 135 µm, which can be challenging for imaging bigger micro-agents. Visual servoing techniques can alleviate this shortcoming by moving the field of view according to the micro-agent motion^125^. Furthermore, a multimodal imaging approach can complement two-photon microscopy and provide additional information on the physical surroundings^126^.

## Discussion

Integrating miniaturized electromagnets into surgical instruments for fluorescence-guided micro-agent manipulation represents a significant advancement in microrobotics. This approach can address the limitations of current electromagnetic systems, particularly in terms of power consumption and spatial constraints. The key strength of this approach is the potential for minimally invasive procedures (e.g., targeted drug delivery and microsurgery) using functionalized micro-agents. However, integrating miniaturized electromagnets into surgical instruments requires careful design and engineering to ensure optimal performance and compatibility. Factors such as electric and thermal insulation and deployment/retrieval mechanisms must be carefully considered to minimize the risk of complications during procedures.

We introduce fluorescence imaging modalities for magnetic micro-agent control, highlighting the micrometer resolution and the capability of obtaining feedback to study biological entities. Fluorescence imaging modalities permit color-coded image acquisition, which is appealing for visualization of micro-agents and physical surroundings without segmentation algorithms. The main drawbacks of conventional fluorescence modalities are the limited penetration depth (< 100 µm) and out-of-focus fluorescence (i.e., background noise).

Two-photon microscopy alleviates the current drawbacks of conventional fluorescence microscopy. However, several challenges remain to be addressed to realize this technology’s potential. One limitation is the penetration depth, which may be insufficient for specific applications when light needs to be focused through thick tissue samples (> 1 mm). Three-photon absorption can improve the penetration depth up to a few millimeters, but the tissue scattering limits the application for thicker samples. An alternative approach involves actively changing the phase and amplitude of the light wavefront (i.e., wavefront shaping). This technique can correct distortions caused by scattering, permitting light to focus through samples up to tens of centimeters. Yet, the triggered fluorescence risks being absorbed by the tissue, hindering its collection for image formation. This motivates the study of fluorescence acquisition in thick samples. Furthermore, the development of endomicroscopy probes integrating high-order photon absorption and wavefront shaping technology holds potential for *in situ* visualization of internal tissues (e.g., mucosa) where required penetration depths are in the order of a few millimeters.

Recognizing the potential of miniaturized electromagnets and two-photon microscopy, we present previous results for fluorescence-guided manipulation of functionalized micro-agents. The results of this synergy showcase the visualization through a rat tissue sample of 600 µm thick. However, further improvements are needed to overcome the scattering effects, relatively low-frame acquisition, and limited field of view. Multimodal imaging strategies, novel scanning techniques, visual servoing, and graphics processing unit parallel computing can optimize the current two-photon microscopy apparatus to be used along with surgical instruments.

## Data Availability

This is a review article, and no new data were generated or analyzed. All data discussed in this article are cited and accessible from the original publications referenced herein.
